# Reporting quality of randomized controlled trial abstracts in the seven highest-ranking anesthesiology journals

**DOI:** 10.1186/s13063-018-2976-x

**Published:** 2018-10-29

**Authors:** Katja Janackovic, Livia Puljak

**Affiliations:** 1 Split-Dalmatia County Pharmacy, Split, Croatia; 2 Cochrane Croatia, University of Split School of Medicine, Soltanska 2, 21000 Split, Croatia; 3 Agency for Quality and Accreditation in Health Care and Social Welfare, Zagreb, Croatia

**Keywords:** Randomized controlled trial, Checklist, Anesthesiology, Abstracts, Reporting, Completeness

## Abstract

**Background:**

The aim of this study was to assess adherence to the Consolidated Standards of Reporting Trials (CONSORT) extension for Abstracts (CONSORT-A) in the highest-impact anesthesiology journals.

**Methods:**

This was a descriptive, cross-sectional, methodological study. We analyzed whether abstracts of randomized controlled trials (RCTs) published in the highest-impact anesthesiology journals between 2014 and 2016 adhered with CONSORT-A. RCT abstracts published in the seven first-quartile journals in the Journal Citation Reports (JCR) category Anesthesiology were analyzed. The primary outcome was adherence to the 17-item CONSORT-A checklist. Secondary outcomes were adherence to individual checklist items and adherence with the checklist across the individual journals.

**Results:**

Search results yielded 688 records, of which 622 abstracts were analyzed. Analysis of the total score of the CONSORT-A checklist indicated a per-article median of 41% (interquartile range 35–53%). The *European Journal of Anesthesiology* had the highest overall adherence rate (53%), whereas *Anaesthesia* had the lowest (32%). The lowest adherence was observed for the following items: Trial design (18%), Contact of the authors as an e-mail address of the corresponding author (16%), Recruitment status (9%), Number of participants analyzed (8%), Randomization (3%), and Funding (0.2%).

**Conclusions:**

RCT abstracts published in top anesthesiology journals are poorly reported, providing insufficient information to readers. Interventions are needed to increase adherence to relevant reporting checklists for writing RCT abstracts.

## Background

Randomized controlled trials (RCTs) enable objective testing of interventions in medicine. When published in a journal, they may not be publicly available in full text due to paywalls, and readers may not be willing or able to pay for reading the full text, but the abstract is often freely available. Therefore, it is important that RCT abstracts are written clearly and with sufficient detail, because readers often use only the abstract as a source of information [1].

Reporting of research manuscripts can be improved by using a relevant checklist that helps authors to report their study in sufficient detail. In order to provide recommendations for reporting RCTs in healthcare journals, the CONSORT (Consolidated Standards of Reporting Trials) Statement was published in 1996 [2] and subsequently updated in 2001 [3]. However, CONSORT provided limited guidance for writing an RCT abstract. Therefore, in 2008 the CONSORT extension for abstracts (CONSORT-A) was published by Hopewell et al. [1]. CONSORT-A is a 17-item checklist whose purpose is to improve reporting of RCT abstracts published both in journals and presented at conferences. Its aim is to “*help authors of abstracts of these trials provide the detail and clarity needed by readers wishing to assess a trial’s validity and the applicability of its results*” [1].

It has been shown recently that reporting of RCT abstracts in high-impact general journals is inadequate [4]. It is important to have clearly reported RCT abstracts across all research fields so that the readers can get a clear picture about the efficacy and safety of an intervention by reading an abstract. We were interested in reporting of RCT abstracts published in the journals from the field of anesthesiology and pain with the highest impact factor, as we hypothesized that even high-impact specialty journals suffer from reporting insufficiencies. Furthermore, we wanted to explore the current state of affairs in more recent years, as CONSORT-A was published ten years ago [1]. The aim of this study was to assess adherence to CONSORT-A in the seven highest-impact anesthesiology journals in the past 3 years.

## Methods

### Study design

This research was a descriptive, cross-sectional, methodological study that analyzed data from published RCTs.

### Inclusion criteria

We analyzed RCTs published between 2014 and 2016 in the first-quartile journals within the Journal Citation Reports (JCR) category Anesthesiology. Seven journals were chosen based on the 2015 JCR impact factor, including (in alphabetical order) *Anaesthesia*, *Anesthesia & Analgesia*, *Anesthesiology*, *British Journal of Anaesthesia*, *European Journal of Anaesthesiology*, *Pain*, and *Regional Anesthesia and Pain Medicine*. We excluded studies that were not RCTs or that did not have an abstract, such as RCTs published as letters to the editor.

### Search

On April 4, 2017 we searched the MEDLINE database via PubMed using a filter for RCTs, a filter for publication dates January 1, 2014 to December 31, 2016, and a journal name. The search syntax was: “Randomized controlled trial[ptyp] AND (“2014/01/01”[PDAT]: “2016/12/31”[PDAT]) AND (“Anesthesiology”[Journal] OR “Pain” [Journal] OR “British journal of anaesthesia”[Journal] OR “ European Journal of Anaesthesiology” [Journal] OR “Anesthesia *&* Analgesia”[Journal] OR “Anaesthesia” [Journal] OR “Regional anesthesia and pain medicine”[Journal])”.

We exported titles and abstracts of retrieved bibliographic records. Two authors independently screened bibliographic records (titles and articles) and excluded those that were clearly not RCTs. In case of doubt, we downloaded full texts in electronic format to judge whether an article was indeed an RCT. Disagreements were resolved via discussion between the authors.

### Outcomes

The primary outcome was proportion of adherence to the CONSORT-A checklist within each article, presented as a median of all articles. Secondary outcomes were overall adherence to CONSORT-A domains and adherence to CONSORT-A across the analyzed journals.

### Scoring the checklist

We designed a data scoring table in a Microsoft Excel spreadsheet (Microsoft Inc., Redmond, WA, USA). We piloted the spreadsheet with five abstracts, and revisions were made before continuing scoring. One author (KJ) independently evaluated all RCT abstracts against the 17 items of CONSORT-A, and the second author (LP) verified all extractions. The reviewers were not blind to manuscript and journal title. The manuscript that described the checklist [1] was reviewed in detail before and during data extraction to ensure a consistent interpretation of each checklist item. Each checklist item was evaluated to analyze whether it was adequately reported, not reported, or unclear in an RCT abstract. We assigned the following scores to each item: ”yes”, ”unclear”, or ”no” to indicate whether it was reported [5]. We resolved discrepancies via discussion.

After the calibration exercise and consideration of potential items that could lead to disagreements, we decide to specify in more detail how certain specific items would be scored. Item #2 (“Contact details for the corresponding author”) was scored ”yes” if the e-mail address of a corresponding author was available. We scored the second item even though CONSORT-A recommended that this item should be used for a conference abstract. We analyzed it in the context of PubMed, as we wanted to see whether the RCT abstract available on PubMed provided the e-mail address of a corresponding author. However, we also provided information on how many abstracts had any affiliation of the authors that could also be considered to be a contact detail.

Item #8 was scored ”unclear” if it was only indicated that the study was randomized but without details about method of randomization; the item was scored ”no” if it was not mentioned in the abstract that the study was randomized. Item #9 was scored ”unclear” if it was only indicated that the study was double-blind but without details about who exactly was blinded (patients, caregivers, outcome assessors). Item #11 was scored ”unclear” if it was not explicitly stated that the study was completed and no longer recruiting. For measuring adherence, we judged responses ”yes” to indicate adherence to the checklist items, while ”unclear” and ”no” were considered as non-adherence to the checklist.

### Data analysis

Descriptive data about RCTs adequately reporting items of the checklist were presented as frequencies and percentages. Adherence per article was reported as median and interquartile range (IQR). Since we analyzed count data, we assumed they were not continuous. For analyses we used MedCalc (MedCalc Software, Mariakerke, Belgium).

## Results

Our search yielded 688 records, of which 622 were eligible. We excluded 66 records because they were not RCTs or they were RCTs published in the form of a letter to the editor and therefore did not have an abstract.

### Primary outcome: adherence to CONSORT-A

Median adherence to CONSORT-A in the analyzed 622 trials was 41% (IQR 35–53%). The majority of analyzed trials (*N* = 442, 70%) had a total adherence score under 50%. Only 11 trials (1.7%) had a total adherence score between 70 and 88%, and not a single trial had total adherence to CONSORT-A that was above 90%.

### Secondary outcome: proportion of trials that adhere to individual items of CONSORT-A

The lowest adherence to CONSORT-A was found for the following items: funding (0.2%), method of randomization (3%), number of participants analyzed in each group (8%), and trial status (9%). The highest adherence was found for the following items: interventions intended for each group, general interpretation of results, detailed results for the primary outcome, and reporting of specific objectives/hypothesis; these domains were reported in more than 90% of the abstracts (Table 1). Poor reporting of the title was very frequent; 39% of the analyzed trials did not indicate in the title that the study was an RCT. The corresponding author e-mail was reported with 16% of the abstracts on PubMed (Table 1). All abstracts were accompanied with author affiliation of a corresponding author, which could also be considered to be “contact details”.

**Table 1:** Adherence to individual items of the CONSORT extension for abstracts

Item	Description	Scored as “yes”, *N* (%)	Scored as “unclear”, *N* (%)	Scored as „no“, *N* (%)
1. Title	Identification of the study as randomized	382 (61)	0	240 (39)
2. Authors	Contact details for the corresponding author	102 (16)	0	520 (84)
3. Trial design	Description of the trial design (e.g., parallel, cluster, non-inferiority)	114 (18)	0	508 (82)
Methods				
4. Participants	Eligibility criteria for participants and the settings where the data were collected	523 (84)	0	99 (16)
5. Interventions	Interventions intended for each group	603 (97)	0	19 (3)
6. Objective	Specific objective or hypothesis	563 (91)	0	59 (9)
7. Outcome	Clearly defined primary outcome for this report	295 (47)	0	327 (53)
8. Randomization	How participants were allocated to interventions	17 (3)	594 (96)	11 (1)
9. Blinding (masking)	Whether or not participants, caregivers, and those assessing the outcomes were blinded to group assignment	302 (49)	0	320 (51)
Results				
10. Numbers randomized	Number of participants randomized to each group	145 (23)	0	477 (77)
11. Recruitment	Trial status	56 (9)	566 (91)	0
12. Numbers analyzed	Number of participants analyzed in each group	48 (8)	0	574 (92)
13. Outcome	For the primary outcome, a result for each group and the estimated effect size and its precision	581 (93)	0	41 (7)
14. Harms	Important adverse events or side effects	128 (21)	0	494 (79)
15. Conclusions	General interpretation of the results	597 (96)	0	25 (4)
16. Trial registration	Registration number and name of trial register	127 (20)	0	495 (80)
17. Funding	Source of funding	1 (0.2)	0	621 (99.8)

There were 25 (4%) of abstracts that did not have a conclusion statement; the majority of those abstracts (*N* = 18) were published in the journal *Anaesthesia*, which had a non-structured abstract and a limit of 150 words for a summary.

### Secondary outcome: adherence to CONSORT-A

The journal *Anesthesia & Analgesia* published the highest number of RCTs in the analyzed period. The highest adherence was found in the *European Journal of Anesthesiology*, where the total adherence score was 53%, while the journal *Anaesthesia* had a total adherence score of 32% (Table 2).

**Table 2:** Number of randomized controlled trials (RCTs) and total adherence to the CONSORT extension for abstracts in different analyzed journals

Journal name	Number of published RCTs	Adherence (%)
*Anaesthesia*	85	32
*Anesthesia & Analgesia*	113	42
*Anesthesiology*	94	44
*British Journal of Anaesthesia*	103	47
*European Journal of Anesthesiology*	67	53
*Pain*	99	43
*Regional Anesthesia and Pain Medicine*	61	43

## Discussion

We found that abstracts of RCTs published in the top anesthesiology journals between 2014 and 2016 have generally poor reporting, as they do not fully adhere to CONSORT-A, which recommends how the title and abstract of an RCT should be reported.

Can et al. compared reporting of RCT abstracts published in four high-ranked anesthesiology journals in 2005–2006 and in 2008–2009 [6] and found that the average adherence was 27% before and 29% after publication of CONSORT-A. This modest increase in average adherence of 2% indicates that the 2008–2009 time period may have been too early for the reporting checklist to become widely known and adopted [6]. Our study shows better compliance compared to the results of Can et al. in the post-CONSORT-A cohort, probably because the awareness of authors, editors, and peer-reviewers about the importance of this checklist has grown over time. Sriganesh et al. analyzed abstracts of five anesthesiology journals in 2005–2007 and 2013–2015; their finding that the mean number of items reported was 6.12 in pre-CONSORT and 7.06 in post-CONSORT-A period indicated also that there is room for improvement [7].

Although one may expect that introduction of a reporting checklist for certain study types will improve reporting, Cui et al. showed that significant improvement in abstracts published in Science Citation Index journals was observed only in three items after introduction of the CONSORT checklist. These three items were trial design, harms, and trial registration [8].

It has been suggested by Hays et al. that incomplete reporting hinders critical appraisal of important studies, and editors were invited to promote reporting checklists as well as to encourage authors to adhere to those checklists [4]. Furthermore, Mbuagbaw et al. suggested that peer-reviewers should also check whether all 17 items of CONSORT-A were properly reported [9].

In this study we found that the median total adherence score per article to CONSORT-A was 41%. Despite the improvement compared to the previous study from the same field [6], the focus should not be on the positive trend but on the observed deficiencies in the quality of RCT abstract reporting. Higher adherence could be expected also because of the fact that the majority of journals analyzed in this study are publicly acknowledged endorsers of the CONSORT statement [10], and all of these journals mention CONSORT in their instructions to authors. Even though none of the analyzed journals mentions CONSORT-A specifically in their instructions to authors of RCTs, the CONSORT checklist specifically indicates in domain 1b for writing an abstract: “for specific guidance see CONSORT for abstracts”.

Our analysis of adherence to individual CONSORT-A items showed extremely poor reporting for several items. Funding was reported in only one of the 622 analyzed abstracts. As it is known that commercial funding may be associated with higher frequency of positive results [11], information about the funder is very relevant to a reader. Furthermore, selective reporting leads to reporting bias and skews the picture about evidence that is available for clinical decision-making [12]. To overcome problems with selective reporting, there is now an imperative for authors to register their RCT protocol in a publicly available clinical trials registry [13]. However, in this study we found that information about clinical trial registration was reported in only 20% of the RCT abstracts.

Furthermore, a clear description of certain methodological elements is crucial for judging risk of bias in RCTs [14, 15]. Can et al. [6] reported that only 1.6% of the analyzed RCT abstracts reported randomization methods, and similar results were also reported by others [16, 17]. Our study found that only 4% of RCT abstracts reported method of randomized sequence generation, indicating that reporting of this item did not improve substantially over the past ten years.

Even though it is easiest to blame the RCT authors for poor reporting, we emphasize that there are some confounding factors that can influence reporting of this part of the manuscript. For example, it could be argued that some of the analyzed journals have a word limit for abstracts that is not compatible with adequate reporting of all the necessary information in the RCT abstract. For example, the journal *Anaesthesia* required abstracts of up to 150 words; the journal *Anesthesia & Analgesia* allowed up to 400 words, while the word limit in the other five journals was 250 words. The CONSORT group considers that 250–300 words in an abstract should be sufficient to properly report all the necessary items [1].

Furthermore, in our analysis we included the second item of CONSORT-A that specifies that “Contact details for the corresponding author” need to be reported, but in CONSORT-A it is recommended that this item is specific for conference abstracts. We analyzed it with particular intention to see for how many RCT abstracts on PubMed there is an available e-mail address of the corresponding author. It is important for readers to have a readily available e-mail address of a corresponding author to foster communication. It is customary to provide the corresponding author’s e-mail address in the full text, but sometimes readers do not have access to the full text because they are not willing or able to pay for it. For this reason we believe that it would be advantageous if journals would provide the e-mail address of the corresponding author when publishing the abstracts on PubMed; in our study only 16% of the analyzed abstracts did so. If one focuses on the broader definition of “contact details”, it is worth emphasizing that all analyzed abstracts had information about the affiliation of a corresponding author. However, we consider it unlikely that someone would try to contact corresponding authors via post.

Our finding that 4% of analyzed abstracts did not have a conclusion statement can be explained with certain journals’ requirement for short abstracts of up to 150 words, as well as the requirement to write unstructured abstracts, which leaves to the authors some liberty regarding organization of information in an abstract. Furthermore, in rare cases authors might have had insufficient academic writing skills, and this lack of conclusion statement was not rectified by peer-reviewers and editors.

Limitations of this study are the limited number of analyzed journals and the limited period in which analyzed trials were published. However, we decided to focus ourselves on a specialty field of our interest, and our hypothesis was that even current RCTs, published long after the introduction of CONSORT-A, suffer from reporting deficiencies. Furthermore, we scored items as “yes”, “no”, and “unclear”, and it is possible that perhaps more scoring categories could give different results.

We might have missed some RCTs by relying exclusively on studies that were indexed as such on PubMed. An alternative methodology would be to hand-search selected journals or to use search options on journal websites; however, different journals have different search functionalities and different pliability to using truncation and adequate search strategies. For this reason, we used PubMed for searching the target studies.

Further studies should analyze interventions for improving adherence to CONSORT-A, including interventions aimed towards authors, editors, and peer-reviewers. Information technologies such as software that checks adherence to reporting guidelines could be a useful solution for insufficient quality of reporting RCTs. In February 2017, the journal *BMJ Open* announced that they have started using Penelope, “an automated online tool that checks scientific manuscripts for completeness and gives immediate feedback to authors “[18]. This automated tool could be customized for various needs, including providing feedback about relevant reporting guidelines that the author might be expected to follow [19]. Using such a tool as an intervention should be tested to see whether it would improve quality of reporting in research manuscripts. We emphasize that a poorly written abstract is not necessarily an indicator of poor research [20], but it certainly deprives a reader of important information about a trial.

## Conclusions

RCT abstracts published in top anesthesiology journals are poorly reported, providing insufficient information to readers. Interventions are needed to increase adherence to relevant reporting checklists for writing RCT abstracts. Innovative software solutions, author education, alerting authors to the relevant checklists in journals’ instructions for authors, and more vigilance from editors and peer-reviewers about this matter could be tested to improve the transparency and reporting of RCT abstracts.

## Abbreviations

CONSORT: Consolidated Standards of Reporting Trials
CONSORT-A: Consolidated Standards of Reporting Trials extension for Abstracts
IQR: Interquartile range
JCR: Journal Citation Reports
RCT: Randomized controlled trial
